# Lipid-Droplet Remodeling in Hepatic Stellate Cells in Liver Fibrosis: An Evidence-Tiered Synthesis of Lipophagy, Retinoid Metabolism, and Organelle Crosstalk

**DOI:** 10.3390/cimb48100988

**Published:** 2026-09-25

**Authors:** Zhe Li, Xinyi Kwan, Qi Liu, Jinwen Chong, Xianjing Jiang, Jingge Wang, Shaodong Chen, Huiqing Liang

**Affiliations:** 1 Department of Traditional Chinese Medicine, School of Medicine, Xiamen University, Xiang’an South Road, Xiang’an District, Xiamen 361102, China; lizhe200212@126.com (Z.L.); kwanxinyi0808@gmail.com (X.K.); 17367965800@163.com (Q.L.); vinyjw28@outlook.com (J.C.); jiangxianjing1@163.com (X.J.); 24520251154873@stu.xmu.edu.cn (J.W.); 2 Center for Liver Disease, Xiamen Hospital of Traditional Chinese Medicine, No. 1739 Xianyue Road, Huli District, Xiamen 361015, China; 3 School of Traditional Chinese Medicine, Xiamen University Malaysia, Jalan Sunsuria, Bandar Sunsuria, Sepang 43900, Selangor Darul Ehsan, Malaysia

**Keywords:** hepatic stellate cell, lipid droplet, liver fibrosis, lipophagy, retinoid metabolism, PNPLA3

## Abstract

In quiescent hepatic stellate cells (HSCs), lipid droplets (LDs) constitute specialized retinoid-rich storage organelles. Upon hepatic injury, various characteristics of LDs—including their abundance, size, composition, retinoid storage capacity, mobilization dynamics, metabolic flux, and associated cell signaling pathways—undergo significant alterations. Current evidence does not support the notion that the visible loss of LDs alone constitutes a uniform or sufficient mechanism driving fibrogenic activation of HSCs. This review distinguishes the machinery that maintains retinoid-rich LD identity from activation-associated lipid mobilization and from organelle relationships that remain primarily functional or inferential. We emphasize the roles of lecithin retinol acyltransferase (LRAT) and retinol-binding protein 1 (RBP1) in retinoid metabolism, compare candidate cytosolic hydrolases with lysosome-dependent lipid mobilization pathways, and differentiate evidence derived directly from HSCs from findings based on broader liver context or general LD biology. We propose an integrated, multidimensional model of HSC LD remodeling, suggesting that therapeutic interventions should target the establishment of a defined, less fibrogenic HSC phenotype rather than merely modulating LD quantity.

## 1. Introduction

Liver fibrosis represents a maladaptive reparative process in which hepatic stellate cells (HSCs) serve as the primary producers of extracellular matrix components. Quiescent HSCs are characterized by the presence of lipid droplets (LDs) enriched with retinoids; however, the activation of HSCs in response to injury cannot be adequately described merely as the disappearance of a single organelle population [1,2,3]. Consequently, the key question is not whether LDs remain visible, but how their composition, storage capacity, mobilization pathways, and metabolic utilization change as the HSC phenotype evolves.

This review addresses five interrelated topics: (1) the mechanisms underlying the establishment of the specialized retinoid-rich state in HSCs; (2) the mobilization pathways for retinoids that have been substantiated by direct functional evidence; (3) the intersection of retinoid handling, lipophagy, and organelle biology; (4) the differentiation between findings derived directly from HSCs versus those inferred from liver-context studies or general lipid droplet biology; and (5) the distinction between associative observations and causal relationships. The organization of this review employs tiers of evidence as an interpretive safeguard rather than as an ultimate evaluative endpoint. The distinctive contribution of this review is its evidence-tiered synthesis: rather than treating all observations on HSC lipid droplets as mechanistically equivalent, we distinguish direct HSC evidence from liver-context evidence and general lipid-droplet biology, separate retinoid storage from lipid mobilization, and identify where organelle-related mechanisms remain inferential. This framework is intended to define causal boundaries and generate experimentally testable predictions.

### Review Scope and Evidence Framework

An initial literature search was conducted using PubMed, Web of Science, and Google Scholar without restrictions on publication date, considering only English-language articles. The initial literature corpus was last updated and screened on 7 July 2026. The primary search terms encompassed hepatic stellate cells (“hepatic stellate cell” OR HSC), lipid droplet biology and metabolism (“lipid droplet,” lipophagy, autophagy, “retinoid metabolism,” PNPLA3, and PPARγ), liver disease contexts (“liver fibrosis,” fibrosis, and MASH), and organelle interactions (“organelle crosstalk,” mitochondria, “endoplasmic reticulum,” and peroxisome). A representative search query was (“hepatic stellate cell” OR HSC) AND (“lipid droplet” OR lipophagy OR “retinoid metabolism” OR PNPLA3) AND (fibrosis OR MASH). During manuscript revision, additional relevant publications were incorporated in response to the reviewers’ comments, particularly those addressing LRAT, RBP1/CRBP-I, retinyl ester metabolism, and lipid-droplet remodeling. This database retrieval was augmented by backward and forward citation tracking of seminal studies pertinent to HSC biology, fibrosis, retinoid metabolism, autophagy, and lipid droplet remodeling.

The evidence was categorized into three tiers: direct HSC evidence, liver-context evidence, and general lipid droplet biology. Direct HSC evidence comprised data obtained from primary HSCs, mechanistically manipulated HSC models, HSC-specific genetic models, human HSCs, or tissue and cell-state analyses resolved at the HSC level. Liver-context evidence included findings from whole-liver injury or fibrosis models and studies involving hepatocytes, macrophages, or multicellular liver systems that establish relevance to liver injury, metabolism, or fibrosis but do not permit attribution of effects specifically to HSCs. General lipid droplet biology encompassed mechanisms of lipid droplet biogenesis, lipid trafficking, autophagy/lipophagy, and organelle interactions primarily elucidated in non-HSC systems. Such studies were employed to support mechanistic plausibility but were not considered definitive evidence of HSC-specific mechanisms.

These tiers function as interpretive categories designed to maintain contextual integrity and causal boundaries; they do not constitute formal assessments of evidence certainty, risk of bias, or meta-analytic rankings. This framework was particularly applied to claims regarding organelle contacts, where findings from other cell types may inform biological plausibility but do not establish mechanisms specific to HSCs.

## 2. HSCs: Physiological Roles and Phenotypic Plasticity

### 2.1. Physiological Roles of Hepatic Stellate Cells

In the healthy liver, HSCs are situated within the space of Disse, positioned between hepatocytes and sinusoidal endothelial cells. This perisinusoidal localization enables HSCs to detect injury-related mediators and facilitates interactions with parenchymal, endothelial, immune, and biliary compartments. Beyond their well-established function in vitamin A storage, HSCs play critical roles in extracellular matrix turnover, regulation of sinusoidal tone and contractility, local growth factor signaling, and tissue repair processes [1,2,4]. These physiological functions provide the essential biological framework within which the specialized lipid droplets of HSCs should be understood.

Under sustained injury, HSCs integrate inflammatory, oxidative, mechanical, and metabolic signals and often acquire a matrix-producing, myofibroblast-like phenotype. This phenotypic shift is characterized by alterations in matrix synthesis, cellular migration, contractility, and secretory profiles, occurring within a complex multicellular hepatic microenvironment. Therefore, changes observed in HSC lipid droplets should be interpreted in the context of concurrent modifications in hepatocyte metabolism, macrophage signaling, and extracellular matrix stiffness, rather than in isolation [1,5,6,7,8].

### 2.2. Quiescent, Activated, and Deactivated Phenotypes

The quiescent phenotype of HSCs is defined by retinoid storage, a lipid-associated transcriptional program, and the maintenance of physiological support functions. Activation of HSCs is marked by increased proliferation, expression of α-smooth muscle actin (α-SMA), enhanced collagen synthesis, and shifts in metabolic demands. Conversely, deactivation or phenotypic reversion can partially restore a less fibrogenic state following injury resolution. While these phenotypic classifications serve as useful experimental frameworks, it is important to note that neither the cellular state nor its reversibility can be conclusively determined based solely on lipid staining.

Seminal studies involving peroxisome proliferator-activated receptor gamma (PPARγ) have demonstrated that activated HSCs can be induced to adopt a transcriptional profile resembling quiescence [9,10]. This evidence underscores a link between adipogenic regulatory pathways and HSC identity; however, it does not imply that an increase in lipid droplet number alone is sufficient to fully restore the quiescent phenotype. This distinction is critical when evaluating lipid droplet reformation, receptor-mediated signaling, and fibrotic outcomes within the same experimental context.

### 2.3. HSC Heterogeneity and Implications for LD Research

HSC populations exhibit considerable heterogeneity influenced by lobular localization, injury models, disease progression, and species differences. Advances in tissue-resolved and single-cell analyses have revealed the coexistence of fibrogenic and inflammatory HSC subpopulations alongside more homeostatic, pericyte-like states [11,12,13,14,15,16,17]. Consequently, retinoid storage capacity and lipid droplet phenotypes may vary among distinct HSC subsets rather than conforming to a singular activation pathway.

This heterogeneity also accounts for the non-equivalence of primary HSCs, culture-activated cells, and established cell lines such as LX-1, LX-2, and HSC-T6. While cell lines offer experimental convenience for perturbation studies, they do not fully replicate the large, retinoid-rich lipid droplet phenotype characteristic of freshly isolated quiescent HSCs [3]. Therefore, it is imperative to adopt a model-aware approach when interpreting data related to lipid storage, mobilization, or organelle interactions in HSC research.

## 3. Lipid Droplets in HSCs: Structural Characteristics, Composition, and Physiological Roles

### 3.1. Fundamental Structure and Biogenesis of Lipid Droplets

LDs are dynamic intracellular organelles characterized by a core of neutral lipids, predominantly triacylglycerols and steryl esters, encased by a phospholipid monolayer rather than a bilayer. LD surface proteins regulate their formation, fusion and fission, accessibility to lipases, intracellular trafficking, and interactions with other organelles [18,19,20,21,22]. This unique structural organization enables LDs to serve as reservoirs that buffer lipid availability while permitting rapid mobilization of stored substrates and lipid species involved in signaling pathways.

The majority of LDs originate from the endoplasmic reticulum (ER), where neutral lipids accumulate between the bilayer leaflets and subsequently bud into the cytosol. This ER origin provides a compelling biological basis for the association of LDs with lipid synthesis and quality control mechanisms. However, the derivation of LDs from the ER does not inherently confirm the existence of a stable or essential tether between LDs and the ER in HSCs. In the context of HSCs, assertions regarding structural contacts necessitate direct visualization or molecular evidence of tethering beyond shared phenotypic responses to ER stress.

### 3.2. Distinctive Features of Lipid Droplets in HSCs

Lipid droplets within HSCs exhibit specialized characteristics, notably the coexistence of retinyl esters alongside triacylglycerols and other neutral lipids, reflecting the role of HSCs as principal hepatic reservoirs of vitamin A [2,4,23]. The composition of these LDs is modulated by dietary retinoid availability and the cellular physiological state, with LD-associated proteins such as perilipins modulating access to the stored lipid pool. In contrast, lipid droplet accumulation in hepatocytes is frequently interpreted as indicative of steatosis and metabolic overload; thus, similar morphological observations bear distinct biological implications in hepatocytes versus HSCs.

The specialized lipid composition of HSC LDs necessitates careful selection of analytical methodologies. While imaging techniques can quantify LD number and size, comprehensive characterization of stored lipid species and their mobilization requires targeted retinoid profiling, neutral lipid lipidomics, and tracer-based approaches. Notably, reductions in bulk fluorescence or dye staining intensity cannot discriminate among retinyl ester depletion, alterations in LD size distribution, substitution by triacylglycerol species, or changes in detection sensitivity.

### 3.3. Remodeling of Lipid Droplets During HSC Activation

Lipidomic and imaging analyses have demonstrated that HSC activation is associated with modifications in LD size distribution and number, accompanied by a replacement of retinyl esters with polyunsaturated triacylglycerol species [24]. Complementary studies reveal that activated HSCs may retain retinoid-containing droplets following transdifferentiation [25]. These findings are not mutually exclusive; rather, they suggest that changes in LD composition, size, and lipid flux occur over distinct temporal scales and may vary across experimental models. A recent study in LX-2 cells activated with transforming growth factor-β1 further showed that oleic-acid-induced partial restoration of intracellular LDs was accompanied by perilipin remodeling but did not significantly reverse fibrotic markers, reinforcing that LD reaccumulation alone is insufficient to establish phenotypic reversion [26].

Consequently, the mere visual depletion of LDs constitutes an incomplete descriptor of HSC phenotypic changes. A more informative approach involves assessing whether perturbations affect retinoid storage capacity, neutral lipid mobilization, downstream substrate utilization, and the fibrogenic transcriptional program collectively. This integrative framework circumvents simplistic models that directly link lipid droplet loss to fibrosis. Figure 1 synthesizes these concepts by situating the specialized lipid droplet phenotype of HSCs within the hepatic sinusoidal microenvironment and across quiescent, activated, and deactivated or reverted cellular states.

## 4. Retinoid Metabolism and the Identity of HSC Lipid Droplets

### 4.1. Intracellular Retinol Handling and the Role of RBP1

Retinol-binding protein 1 (RBP1; also known as CRBP-I) plays a critical role in the retention and directed metabolism of retinol within cells. Its mechanistic significance in HSC biology lies in its capacity to enhance the efficiency of retinol esterification by facilitating retinol availability to lecithin retinol acyltransferase (LRAT), while concurrently restricting unregulated access to competing metabolic pathways. Studies involving CRBP-I-deficient mice reveal diminished accumulation of retinyl esters in HSCs, attributable to both decreased synthesis and increased turnover, rather than a direct profibrotic transformation [27].

Further cellular investigations corroborate that CRBP-I expression is responsive to extracellular retinol levels and correlates with cytosolic retinol-binding capacity in HSC models [28]. These findings align with a functional role in intracellular retinol handling and storage. However, it is important to emphasize that these genetic and in vitro studies were primarily designed to elucidate retinoid homeostasis and should not be interpreted as evidence that RBP1 functions as an antifibrotic effector.

### 4.2. Retinyl Ester Synthesis and LRAT-Dependent Storage

LRAT is the principal physiological enzyme responsible for retinyl ester synthesis in the liver and is central to the characteristic retinoid-rich LD phenotype of HSCs. The critical role of LRAT is demonstrated in vivo, as *Lrat* knockout mice lack the classical retinoid-containing lipid droplets in HSCs [29,30]. In contrast, primary HSCs isolated from *Lrat*-null mice retain small neutral lipid droplets and exhibit limited retinyl ester synthesis ex vivo, mediated through alternative acyl-CoA-dependent esterification pathways, including diacylglycerol O-acyltransferase 1 (DGAT1)-associated activity [30]. This discrepancy between in vivo and ex vivo observations delineates the classical LRAT-dependent storage phenotype from residual neutral lipid and retinyl ester synthesis capacity, cautioning against inferring HSC activation solely from the absence of classical retinoid droplets.

This distinction is critical for accurate interpretation. In *Lrat*-deficient mice, the absence of retinoid-rich lipid droplets in HSCs did not exacerbate fibrosis following bile duct ligation (BDL) or carbon tetrachloride (CCl4)-induced injury, nor did it promote spontaneous fibrosis [29]. These findings indicate that loss of classical retinoid-rich LDs alone does not cause spontaneous fibrosis or exacerbate fibrosis in the injury models tested.

### 4.3. Retinyl Ester Mobilization

Hormone-sensitive lipase (HSL; encoded by *LIPE*) has been identified as a direct hydrolase of retinyl esters in quiescent human and rat HSCs, representing one of the more clearly characterized cytosolic enzymes involved in retinyl ester mobilization [31]. Additionally, KIAA1363 has been shown to modulate retinyl ester turnover in cultured murine and human HSCs [32]. While these studies demonstrate enzymatic activity or perturbation effects within specific experimental models, they do not establish a universally rate-limiting hydrolase responsible for retinyl ester mobilization across intact liver injury contexts.

The patatin-like phospholipase domain-containing protein 3 (PNPLA3) introduces a genotype-dependent dimension to retinoid metabolism. The wild-type PNPLA3 exhibits retinyl-palmitate lipase activity in human HSCs, whereas the I148M variant significantly diminishes this enzymatic function [33]. Complementary studies employing human HSCs and three-dimensional matrix models indicate that the I148M variant alters retinoid handling and is associated with a more fibrogenic cellular phenotype [34,35]. Consequently, the evidence supports a genotype-conditioned modulation of lipid metabolism rather than a fixed, context-independent role for PNPLA3.

Adipose triglyceride lipase (ATGL; encoded by *PNPLA2*) and its coactivator comparative gene identification-58 (CGI-58; also known as ABHD5) have been localized to lipid droplets in HSC-T6 cells, suggesting the presence of a lipid droplet-resident neutral lipid mobilization system [36]. However, given that these observations derive from a cell line model and do not constitute direct evidence of retinyl ester hydrolase activity in primary HSCs, ATGL should be considered separately from HSL, KIAA1363, and PNPLA3. 

### 4.4. Retinoid-Sensitive Nuclear Receptor Signaling and HSC Identity

Pathways associated with PPARγ and retinoid X receptor (RXR) primarily regulate transcriptional programs and cellular states rather than directly mediating the enzymatic synthesis of retinyl esters. Restoration of PPARγ expression has been shown to shift activated HSCs toward a less activated phenotype. Moreover, adipogenic and ETS proto-oncogene 1 (ETS1)–CREB-regulated transcription coactivator 2 (CRTC2)/PPARγ coactivator 1α (PGC-1α)–PPARγ signaling programs are correlated with the maintenance of quiescence-associated cellular identity [9,10,37,38]. Experimental evidence demonstrates that re-expression of perilipin 5 (PLIN5), as well as its induction via curcumin treatment, can promote the reformation of LDs while concurrently attenuating HSC activation [39,40]. This suggests that restoration of LDs may accompany broader phenotypic transitions, although it does not establish LD quantity as the direct causal factor. Consequently, proteins such as RBP1 and LRAT are primarily implicated in retinoid handling and storage processes, whereas PPARγ and RXR provide the signaling framework through which variations in retinoid availability may modulate HSC state.

This conceptual distinction also informs the interpretation of receptor-targeted interventions. Therapeutic approaches that modify PPARγ-responsive transcriptional activity and activation markers may influence LD organization as part of a comprehensive cellular state transition; however, such changes do not imply that PPARγ functions as an enzyme responsible for retinyl ester storage. Conversely, replenishing retinyl esters without reinstating the associated transcriptional program does not necessarily restore functional quiescence.

### 4.5. Implications of Retinoid Loss in HSC Activation

Retinoid loss commonly accompanies HSC activation, but this change alone neither defines the activated phenotype nor establishes a causal role in activation. Activated HSCs may retain retinoid-containing lipid droplets, and retinyl esters can be substituted by other neutral lipid species rather than being completely eliminated from all droplets [24,25].

The *Lrat*-deficient findings summarized in Section 4.2 constrain causal inference: loss of the classical retinoid storage compartment alone did not increase fibrosis in the settings tested [29]. This does not exclude injury-specific roles for retinoid metabolism.

Furthermore, nuclear receptor-mediated programs introduce an additional layer of complexity: retinoid metabolism, retinoid-responsive signaling, and regulation of cellular state may co-occur without being functionally interchangeable. Collectively, the data support the interpretation that loss of retinoid-rich lipid droplets constitutes a context-dependent component or biomarker of cellular remodeling, rather than serving as an autonomous causal determinant of HSC phenotype. Table 1 reflects this distinction by categorizing candidate enzymes according to their physiological relevance rather than equating their functional roles.

## 5. Lipophagy and Lipid Mobilization During HSC Activation

### 5.1. Autophagy-Dependent Lipid Mobilization

The most compelling direct functional evidence for lipid mobilization during HSC activation derives from studies on autophagy. Hernández-Gea et al. demonstrated that autophagy facilitates the release of stored lipids and promotes fibrogenesis in activated HSCs within both murine and human tissue contexts. Independently, Thoen and colleagues corroborated the involvement of autophagy in HSC activation [43,44]. These investigations link autophagic flux to the availability of intracellular lipid substrates, rather than merely reflecting alterations in LD morphology.

A plausible functional interpretation is that the mobilization of stored lipids supplies substrates necessary to meet the energetic and biosynthetic demands associated with HSC activation. This interpretation is supported by the activation-specific context of the direct HSC studies; however, it should not be overgeneralized to assert that all activated HSCs derive their oxidative substrates exclusively from LD degradation. The causal conclusion is narrower: autophagy-dependent lipid mobilization can support activation in the tested models.

Furthermore, the role of autophagy must be contextualized according to cell type, substrate specificity, and disease stage. While direct studies on HSCs support activation-associated autophagy and lipophagy [43,44], broader literature on liver disease indicates that the relationship between autophagy and LDs varies across hepatic cell populations and pathological contexts [45,46]. Consequently, apparent pro- or antifibrotic outcomes are not directly comparable unless the cellular compartment and functional endpoints are explicitly defined.

### 5.2. Selective Lipophagy and Rab18

Research focusing on Rab18 extends the understanding from bulk autophagy toward selective LD trafficking and lipophagic flux. Experimental perturbations of Rab18-related trafficking, phase behavior, or LD-associated functions have been shown to modulate lipophagic flux and antifibrotic responses in HSC models [41,42]. These findings position Rab18 as an emerging candidate regulator of selective lipid mobilization rather than a universal mechanism operative in all contexts.

However, the current evidence base remains limited in scope and context. It remains unclear whether Rab18 functions similarly across primary HSCs, established HSC lines, and diverse fibrosis etiologies, or whether its effects represent a common upstream stress response. Therefore, Rab18 should be regarded as an emerging, evidence-constrained regulator of selective lipid droplet mobilization rather than a broadly applicable mechanism in HSC biology.

### 5.3. Cytosolic Lipolysis Versus Lysosomal Mobilization

Cytosolic and lysosomal pathways address related but distinct aspects of lipid mobilization. Enzymes such as HSL, KIAA1363, and PNPLA3 have been primarily investigated as candidate retinyl ester hydrolases or as genotype-dependent modulators of lipid handling in HSCs, whereas ATGL is chiefly implicated in neutral lipid mobilization within HSC-T6 cell models [31,32,34,35,36,47]. In contrast, autophagy and lipophagy studies focus on lysosome-dependent access to stored lipids and exhibit stronger direct functional associations with HSC activation [43,44].

These pathways differ in terms of cellular compartments, lipid substrates, and experimental interventions: enzyme-focused studies typically employ activity assays, genetic perturbations, or specific cell models to interrogate particular lipid species, whereas autophagy investigations manipulate autophagic flux and assess activation-related outcomes. It is therefore inappropriate to conflate these mechanisms into a singular linear pathway. They may operate concurrently or under distinct physiological conditions; however, evidence for compensatory interactions between cytosolic and lysosomal routes remains lacking.

Accordingly, current data support an evidence-tiered model rather than a monolithic mechanism. Lysosome-dependent lipid mobilization possesses the most robust functional evidence in HSCs, while cytosolic candidates provide more specialized, model- and substrate-dependent insights. This distinction underlies the rationale for Table 1’s differentiation of mechanism, experimental model, and limitations, rather than presenting all lipid droplet regulators as functionally equivalent.

### 5.4. Metabolic Implications of Lipid Droplet Mobilization

The mobilization of LDs can alter fatty acid availability, membrane remodeling processes, and oxidative stress levels, particularly during periods when activated HSCs exhibit elevated energetic and biosynthetic demands [5,6]. Inhibition of carnitine palmitoyltransferase 1A (CPT1A) within HSCs has been shown to confer protection against fibrosis, thereby providing direct evidence for the involvement of fatty acid oxidation (FAO) in the metabolic reprogramming associated with HSC activation [48]. Additional independent investigations into HSCs, focusing on the loss of carcinoembryonic antigen-related cell adhesion molecule 1 (CEACAM1), acetate and cholesterol synthesis, lactate transport, and the p63–human epidermal growth factor receptor 2 (HER2)–acetyl-CoA carboxylase 1 (ACC1) signaling axis, further corroborate that fibrogenic activation is embedded within a broader context of metabolic remodeling [49,50,51,52]. Consequently, mitochondrial and redox adaptations can be functionally associated with the state of LD mobilization, although this does not necessarily imply that all such metabolic pathways are downstream effects of LD degradation.

It remains critical to delineate the boundaries of causality in this context. While evidence supports the participation of FAO in HSC activation, this does not establish that all FAO substrates originate exclusively from LD degradation, nor that every mitochondrial alteration is a consequence of lipophagy. Other factors, including parallel injury signals, extracellular lipid sources, and alternative intracellular lipid stores, may also contribute to these metabolic changes. The most substantiated conclusion is that LD mobilization potentially influences metabolic adaptation by modulating substrate availability. Figure 2 integrates the processes of retinoid handling and storage with both cytosolic and lysosome-dependent mobilization pathways, underscoring the necessity of interpreting lipid droplet remodeling in conjunction with the overall HSC activation state rather than considering it as an isolated marker of activation.

## 6. Organelle Crosstalk in HSC Lipid-Droplet Remodeling

### 6.1. Lipid Droplet–Lysosome Interactions

The biological basis for interactions between LDs and lysosomes is most compelling in contexts where autophagic machinery mediates the delivery of stored lipids to degradative compartments. In HSCs, functional evidence indicates that disruption of autophagy affects lipid mobilization and outputs associated with cellular activation [43,44], thereby supporting the role of lysosome-dependent processing as a functional component of lipid-droplet remodeling in these cells. It is important to distinguish functional mobilization from the existence of stable structural contact sites between LDs and lysosomes. While functional interactions can be demonstrated through flux and perturbation experiments, confirmation of stable contact sites requires imaging techniques capable of resolving organelle contacts or molecular evidence of tethering. Current research in HSCs more robustly supports the functional aspect, whereas broader studies in lipid-droplet biology provide contextual support for potential organelle apposition. 

### 6.2. Lipid Droplet–Mitochondria Interactions

Coordination between LDs and mitochondria is biologically plausible given that fatty acids liberated from intracellular lipid stores can fuel mitochondrial metabolism, and activated HSCs exhibit significant metabolic reprogramming. Direct investigations in HSCs have demonstrated that CPT1A-dependent fatty acid oxidation and AMP-activated protein kinase (AMPK)–mitophagy-mediated mitochondrial adaptations contribute to phenotypes associated with activation [48,53]. These findings underscore the importance of mitochondrial metabolism and quality control in HSC activation; however, they do not confirm that the substrates involved are directly supplied from lipid droplets, nor do they establish the necessity of a physical LD–mitochondria interface. Studies conducted in the broader hepatic context reveal that mitochondrial dysfunction and metabolic stress are features of fibrotic liver disease [54,55], but these observations cannot be specifically attributed to LD–mitochondria coupling within HSCs. While structural contacts between LDs and mitochondria are well documented in other cell types [56], evidence demonstrating equivalent tethering, contact-resolved imaging, or the functional requirement of such contacts in HSCs remains insufficient. Consequently, the strongest HSC-specific evidence pertains to mitochondrial metabolic function and adaptation, whereas direct lipid transfer from LDs to mitochondria and the necessity of structural LD–mitochondria contacts remain unproven.

### 6.3. Lipid Droplet–Endoplasmic Reticulum Interactions

The ER plays a central role in lipid-droplet biogenesis across cell types, rendering it a critical mechanistic context for lipid-droplet remodeling in HSCs. In HSCs, direct studies have implicated ER stress in fibrogenic activation and have identified ER-associated degradation (ERAD) pathways as contributors to the restoration of lipid homeostasis [57,58]. These findings establish the relevance of ER stress and lipid homeostasis responses in HSCs but do not provide evidence for a direct LD–ER contact mechanism specific to these cells. General cellular studies have demonstrated that lipid droplets originate from and maintain functional coupling with the ER [18,19,20,21,22]. However, HSC-specific investigations have yet to identify a requisite LD–ER tethering complex or to demonstrate that physical LD–ER contacts are essential for fibrogenic activation. Therefore, current evidence supports an association between ER stress and lipid homeostasis in HSCs, with strong plausibility derived from general cell biology for LD–ER coupling, while direct evidence for contact-site dependence in HSCs remains to be established.

### 6.4. Relationships Between Lipid Droplets and Peroxisomes

Peroxisomes are generally recognized for their potential role in lipid metabolism within the broader context of LD biology, as evidenced by well-characterized peroxisomal membrane contact sites in mammalian cells [59]. However, direct evidence specific to HSCs remains limited. Therefore, the appropriate interpretation is not the absence of an LD–peroxisome interaction mechanism in HSCs, but rather that this area remains preliminary and necessitates targeted, cell-specific structural and functional investigations before such mechanisms can be integrated into causal frameworks. Figure 3 provides a summary of the evidence-tiered interpretation of LD interactions with lysosomes, mitochondria, ER, and peroxisomes, differentiating between established functional associations and structural contact-site claims that have yet to be validated. These findings support an evidence-tiered interpretation of LD interactions with lysosomes, mitochondria, ER, and peroxisomes, as summarized in Table 2.

## 7. An Integrated Evidence-Tiered Framework for HSC Lipid-Droplet Remodeling

LD remodeling in HSCs should be conceptualized as a coordinated alteration encompassing multiple parameters, including LD abundance, size, lipid composition, retinoid storage capacity, mobilization and flux, metabolic utilization, and the associated transcriptional landscape. This comprehensive definition acknowledges observations that activated HSCs may retain LDs while modifying their content and spatial distribution [24,25], thereby moving beyond a simplistic binary, image-based presence-or-absence paradigm. Figure 1 contextualizes this multidimensional definition by illustrating relevant phenotypic and lipid-droplet remodeling features.

While storage and mobilization are interrelated processes, they represent distinct dimensions. Proteins such as RBP1 and LRAT facilitate retinol handling and classical retinoid-rich storage [27,28,30]. By contrast, HSL, KIAA1363, PNPLA3, and ATGL, together with autophagy-related pathways, have been implicated in lipid mobilization, although their substrates and supporting evidence differ [31,32,34,35,36,43,44,47]. Notably, findings from *Lrat*-deficient models demonstrate that abrogation of classical retinoid storage alone did not exacerbate fibrosis in the injury models tested, underscoring the complexity of these pathways [29]. Figure 2 integrates these storage and mobilization aspects while maintaining clear distinctions regarding the strength of supporting evidence.

Taken together, the organelle evidence is asymmetric: lysosome-dependent lipid mobilization has direct functional support in HSCs, whereas mitochondrial and ER findings primarily establish metabolic and homeostatic relationships. The LD–peroxisome axis remains exploratory. These distinctions are summarized in Figure 3 and Table 2 and should not be interpreted as evidence of validated HSC-specific structural contacts.

This integrated model thus enables a more nuanced causal interpretation. Although the loss of retinoid-rich LDs may coincide with HSC activation, it neither constitutes a sufficient phenotypic marker nor a standalone causal mechanism. Robust claims that specific LD pathways drive HSC states require coordinated perturbation experiments, precise lipid-species quantification, and functional assessments resolved by cellular state.

## 8. Disease Context and HSC Heterogeneity

### 8.1. MASLD/MASH

Metabolic dysfunction-associated steatotic liver disease (MASLD) and metabolic dysfunction-associated steatohepatitis (MASH) represent critical contexts wherein hepatocyte lipid accumulation and HSC LD remodeling occur concurrently within the injured liver but should not be conflated. Hepatocyte lipid storage primarily reflects steatosis and metabolic stress, whereas alterations in HSC LDs must be interpreted in relation to retinoid storage, cellular activation, and extracellular matrix [7,8,11,12,60,61,62,63]. The broader MASH literature emphasizes the interplay among metabolic, inflammatory, and fibrotic pathways [8,61,64], with disrupted vitamin A metabolism in non-alcoholic fatty liver disease providing additional retinoid-related context [65]. This distinction cautions against the inappropriate extrapolation of hepatocyte LD mechanisms to HSC biology.

Human multimodal and single-cell analyses have revealed heterogeneous HSC programs in MASH, offering opportunities to link LD-associated phenotypes with clinically relevant cellular states [11,12,13,14,15,66]. Additionally, senescent HSC phenotypes represent disease-stage-dependent programs implicated in MASH-associated inflammation and progression [67]. While these datasets enhance translational relevance, they do not substitute for mechanistic perturbation studies. Therefore, integrating tissue-resolved profiling with detailed lipidomic analyses and functional HSC models remains essential.

### 8.2. Alternative Contexts of Liver Injury

Liver injuries associated with alcohol consumption, viral infections, cholestasis, and toxic insults each activate HSCs within unique inflammatory, oxidative, and metabolic milieus. Consequently, LD-associated mechanisms observed in these disease contexts should not be assumed to generalize across etiologies [4,68,69]. These diverse contexts are more appropriately utilized to assess the external validity of findings rather than to suggest the existence of a singular, shared LD pathway.

Studies conducted within specific disease contexts warrant independent interpretation. For instance, in experiments involving LX-2 cells and murine models, exposure to nonylphenol induced PTEN-induced kinase 1 (PINK1)/Parkin-mediated mitophagy, which was accompanied by the loss of PLIN5, degradation of lipid droplets, and markers indicative of fibrosis [70]. Another investigation focusing on HSCs demonstrated that exposure to human immunodeficiency virus (HIV) R5/gp120 correlated with lipid droplet depletion and a profibrotic phenotype [71]. Additionally, in a toxic-exposure model, taurine was found to mitigate arsenic trioxide (As_2_O_3_)-induced HSC activation via a peroxisome proliferator-activated receptor alpha (PPARα)-autophagy signaling pathway [72]. In an inorganic-arsenic exposure model, PLIN5 was implicated in HSC lipophagy, but its direct contribution to arsenic-induced HSC activation was not established [73]. It is important to emphasize that these findings are specific to their respective models and do not establish a universal mechanistic pathway linking disease-induced stress, lipid droplet loss, and fibrosis.

### 8.3. Human Variability, PNPLA3 Genotype, and Model Dependence

The use of primary human HSCs and three-dimensional extracellular matrix (ECM) models is particularly critical for investigating genotype-dependent pathways. The PNPLA3 I148M variant influences retinoid metabolism within HSCs and modulates their fibrogenic phenotype. Moreover, three-dimensional models reveal how the ECM context can alter the interpretation of cell-autonomous effects [34,35,47]. Such approaches provide more robust evidence for human relevance compared to extrapolations derived from a single immortalized cell line; however, they still necessitate validation across multiple donors, disease stages, and etiologies.

Advanced methodologies, including single-cell and spatial transcriptomic analyses, enable the identification of distinct HSC subpopulations. Meanwhile, precision-cut liver slices and induced pluripotent stem cell (iPSC)-derived HSC-like models facilitate the preservation or reconstruction of multicellular microenvironments. Therefore, the critical question regarding external validity extends beyond the mere detection of a pathway in a human-relevant system; it concerns whether the pathway remains consistently associated with lipid droplet composition and the functional state of HSCs across pertinent subpopulations.

## 9. Therapeutic Implications

Therapeutic strategies should prioritize inducing a defined HSC phenotype characterized by reduced fibrogenic activity, rather than merely modulating the quantity of LDs. Current therapeutic approaches targeting HSCs encompass receptor modulation, metabolic regulation, autophagy manipulation, and cell-specific interventions [74]. Although retinoid metabolism represents a mechanistic target, findings from *Lrat*-deficient models caution against considering restoration of the classical lipid storage phenotype as a validated antifibrotic endpoint [29]. Moreover, interventions that operate via pathways independent of LD dynamics, such as direct inhibition of transforming growth factor-beta signaling within HSCs, underscore the necessity of evaluating antifibrotic efficacy based on functional outcomes rather than LD abundance alone [75]. Consequently, effective therapeutic programs should integrate assessments of lipid state with fibrosis-relevant HSC functional parameters.

Restoration of the PPARγ-associated phenotype is supported by experimental evidence, given that PPARγ activation can reprogram activated HSCs toward a quiescent-like state [9,10,38]. Investigations focusing on HSC-specific ETS1 further corroborate the involvement of an ETS1–CRTC2/PGC-1α–PPARγ axis associated with quiescence [37]. In contrast, the use of PPARα/γ agonists such as saroglitazar within the hepatic context should be interpreted as indicative of broader metabolic effects rather than direct evidence of HSC LD restoration [76]. Therefore, therapeutic interpretations should extend beyond LD restoration alone, recognizing that receptor signaling may concurrently influence extracellular matrix production, inflammatory responses, and metabolic processes alongside LD organization.

Targeting lipophagy and lysosome-dependent lipid mobilization represents a mechanistically grounded approach, as direct studies in HSCs have linked autophagy-mediated lipid release to cellular activation [43,44]. However, broad modulation of autophagy is not inherently beneficial, given the differential responses of hepatocytes, macrophages, and HSCs, as well as the dependency on disease stage and substrate specificity. Compounds such as diallyl trisulfide, curcumol, and honokiol serve as representative preclinical probes targeting Rab18/lipophagy or lysosomal pathways, though they have yet to be validated clinically [41,42,77].

PNPLA3 offers a genotype-informed therapeutic avenue, as the I148M variant is associated with altered lipid handling in human HSCs [33,34,35,47,78]. Additional targets include mitochondrial metabolism and CPT1A-mediated FAO, although interventions may exert distinct effects on hepatocytes versus HSCs [48,53,54]. Accordingly, the appropriate translational endpoint should be defined as a cell-type-specific improvement in fibrosis rather than changes in isolated metabolic markers.

ER stress and lipid homeostasis pathways constitute another preclinical target class. Agents such as arctigenin and oroxylin A exemplify experimental probes of ERAD-linked homeostasis and ATGL-mediated lipid droplet turnover, respectively [57,79]. Nonetheless, natural products frequently modulate multiple stress and metabolic pathways simultaneously, a pleiotropic effect that is well-documented in the broader literature on MASLD and MASH [80]. Thus, their utility currently lies in mechanistic hypothesis generation pending selective, human HSC-specific, and clinical validation.

Across all therapeutic classes, selective delivery to HSCs is likely essential. Systemic modulation of autophagy, nuclear receptors, or metabolism may yield beneficial effects in one hepatic cell type while adversely affecting others, and fibrosis resolution can also be indirectly modulated via restorative macrophage programs [81]. Therefore, ligand-directed or nanotherapeutic delivery systems should be evaluated using cell-type-specific safety assessments, genotype-informed designs, and established fibrosis models rather than relying solely on short-term cell culture data. Table 3 categorizes these mechanism-based therapeutic strategies according to their translational stage, mechanistic rationale, evidence basis, and principal limitations.

## 10. Methodological Challenges and Prospective Research Directions

A primary methodological imperative involves differentiating physical organelle contact from mere functional association. Concurrent stress responses may induce parallel alterations in LDs, lysosomes, mitochondria, and the ER without necessarily indicating the presence of bona fide contact sites. To rigorously evaluate whether proposed inter-organelle interfaces exist, exhibit dynamic behavior, and are essential within primary HSCs, a combination of live-cell imaging, electron microscopy, correlative methodologies, organelle-specific reporters, and proximity labeling techniques should be employed.

The second critical priority pertains to the causal dissection of discrete LD-associated pathways. Experimental designs should independently perturb processes such as lipophagy initiation, lysosomal flux, candidate retinyl ester hydrolase activities, PNPLA3 enzymatic function, PLIN5-mediated LD re-formation, β-oxidation, mitophagy, and ERAD. Corresponding outcome measures must encompass lipid composition, retinoid species profiles, activation markers, collagen synthesis, cell viability, and extracellular matrix behavior to ensure that alterations in a single parameter are not erroneously interpreted as indicative of a comprehensive phenotypic transition.

Lipid specificity is essential. Microscopic analyses alone are insufficient to determine which lipids are mobilized from HSC LDs, their subsequent intracellular destinations, or their functional roles as metabolic substrates, signaling ligands, membrane precursors, or stress mediators. Integrative approaches combining retinoid profiling, lipidomics, isotope tracing, and temporally resolved imaging are necessary to discriminate retinyl ester turnover from triacylglycerol remodeling and to ascertain whether metabolic adaptations are consequent to lipid mobilization or occur concomitantly.

Ensuring translational relevance to human physiology necessitates the utilization of models that maintain cellular heterogeneity and extracellular context. These include primary human HSCs, three-dimensional ECM cultures, iPSC-derived HSC-like cells, precision-cut liver slices, and spatially resolved single-cell methodologies. Stratification by PNPLA3 genotype, disease stage, etiology, and HSC subpopulation identity within these systems is essential. Given that these models are not interchangeable, their complementary application can delineate conserved phenomena from model-specific observations.

### Experimentally Testable Predictions Informed by the Integrated Model

The integrated model yields three empirically testable hypotheses. First, if remodeling of HSC lipid droplets is predominantly characterized by alterations in lipid composition and flux rather than by uniform droplet disappearance, then measurements of lipid species and flux should correlate more robustly with HSC activation status than assessments of lipid droplet abundance alone. This hypothesis can be evaluated by integrating lipid droplet imaging with retinoid profiling, neutral lipid lipidomics, isotope tracing, and matched HSC activation markers such as α-SMA, COL1A1 expression, and collagen production.

Second, if lysosome-dependent lipid mobilization functionally facilitates HSC activation, then selective perturbation of lipophagy or lysosomal flux should modulate fibrogenic outcomes concomitant with substrate mobilization, rather than merely altering the visible number of lipid droplets. Notably, broad autophagy modulation within heterogeneous liver cell populations would be insufficient to rigorously test this prediction.

Third, if the hypothesized functional relationships between LDs and mitochondria or between LDs and the ER are mechanistically required rather than parallel manifestations of cellular stress, then disruption of the pertinent physical or functional interfaces should impact both organelle-associated lipid metabolism and HSC activation. Testing this hypothesis requires the contact-resolving approaches outlined in Section 10, paired with targeted perturbation of candidate tethering proteins in HSC-specific systems.

These predictions are intended to render the evidence-based model experimentally falsifiable and do not imply that the proposed mechanisms have been definitively established.

## 11. Conclusions

LD remodeling in HSCs is complex and multidimensional, rather than simply involving a uniform disappearance of LDs. The quantity of LDs alone does not determine the HSC phenotype; it is essential to separately assess retinoid storage and lipid mobilization. Loss of retinoid-rich LDs alone is insufficient to establish fibrogenic activation, whereas lysosome-dependent lipid mobilization has relatively strong direct functional support in HSCs. Structural LD–mitochondria, LD–ER, and especially LD–peroxisome mechanisms remain incompletely demonstrated in HSCs. In summary, an evidence-tiered interpretation shifts the conceptual focus from the disappearance of HSC lipid droplets to the coordinated remodeling of lipid identity, storage, mobilization, organelle interactions, and cellular phenotype. This framework may provide a more precise basis for identifying therapeutically actionable mechanisms in HSC biology.

## Figures and Tables

**Figure 1:**
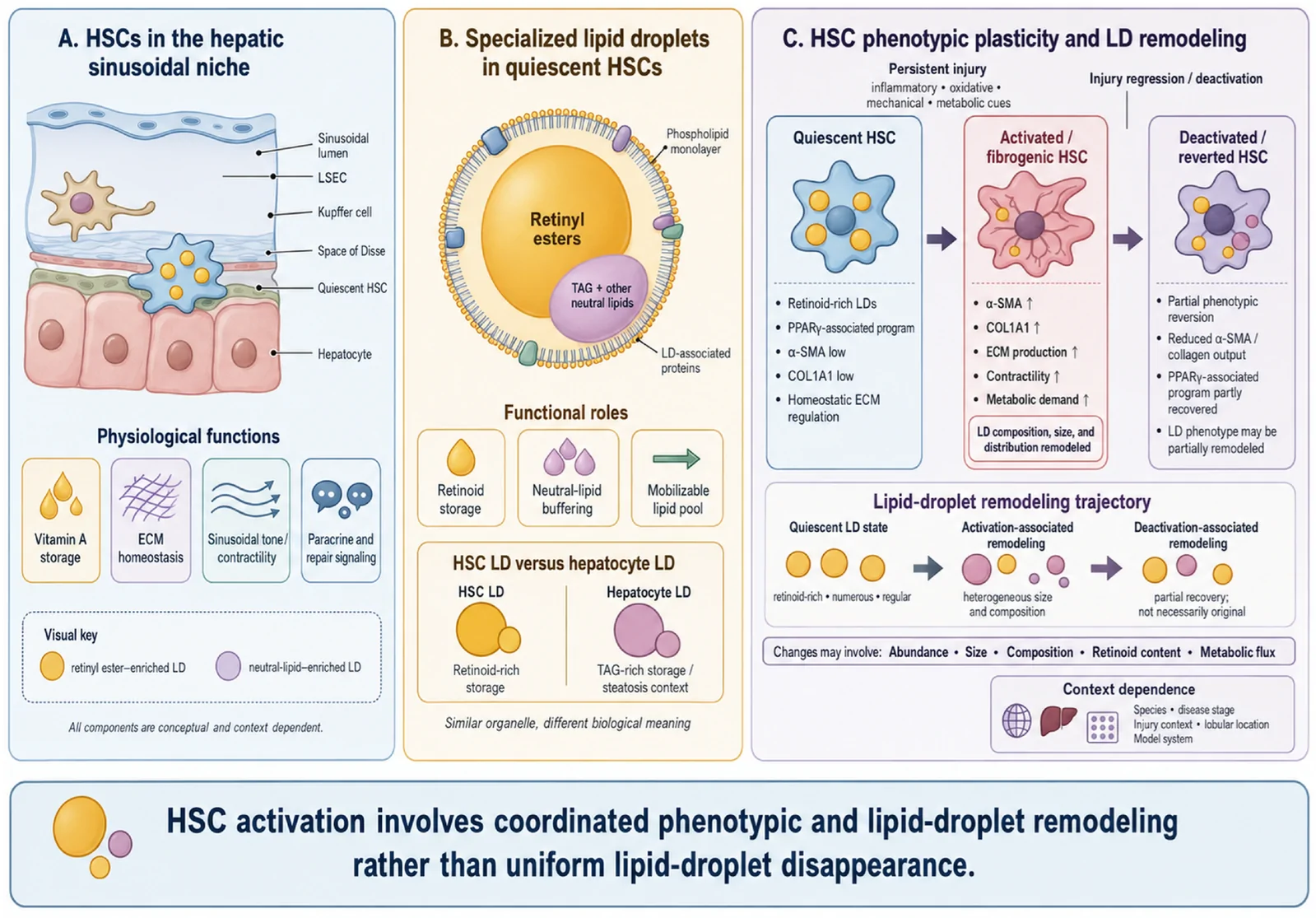
Phenotypic plasticity of hepatic stellate cells and the specialized remodeling of lipid droplets. (**A**) In their quiescent state, hepatic stellate cells (HSCs) are located within the space of Disse, where they contribute to vitamin A storage, maintenance of extracellular matrix homeostasis, regulation of sinusoidal tone and contractility, as well as local paracrine signaling and tissue repair processes. (**B**) The lipid droplets within HSCs comprise retinyl esters alongside triacylglycerols and other neutral lipids, organized within a neutral lipid core encased by a phospholipid monolayer; this function in retinoid storage is distinct from the lipid droplet accumulation associated with steatosis in hepatocytes. (**C**) Chronic hepatic injury induces HSC activation, whereas resolution of injury may allow for partial deactivation or reversion to a quiescent-like state. Throughout these transitions, the abundance, size, composition, retinoid content, and metabolic dynamics of lipid droplets may undergo remodeling, without necessarily resulting in their complete disappearance. Purple arrows indicate transitions between HSC phenotypic states associated with persistent injury or injury regression/deactivation, respectively, and do not imply a single linear or causally established molecular pathway. Upward arrows indicate increased expression or activity. Created using ChatGPT (OpenAI, GPT-5.6).

**Figure 2:**
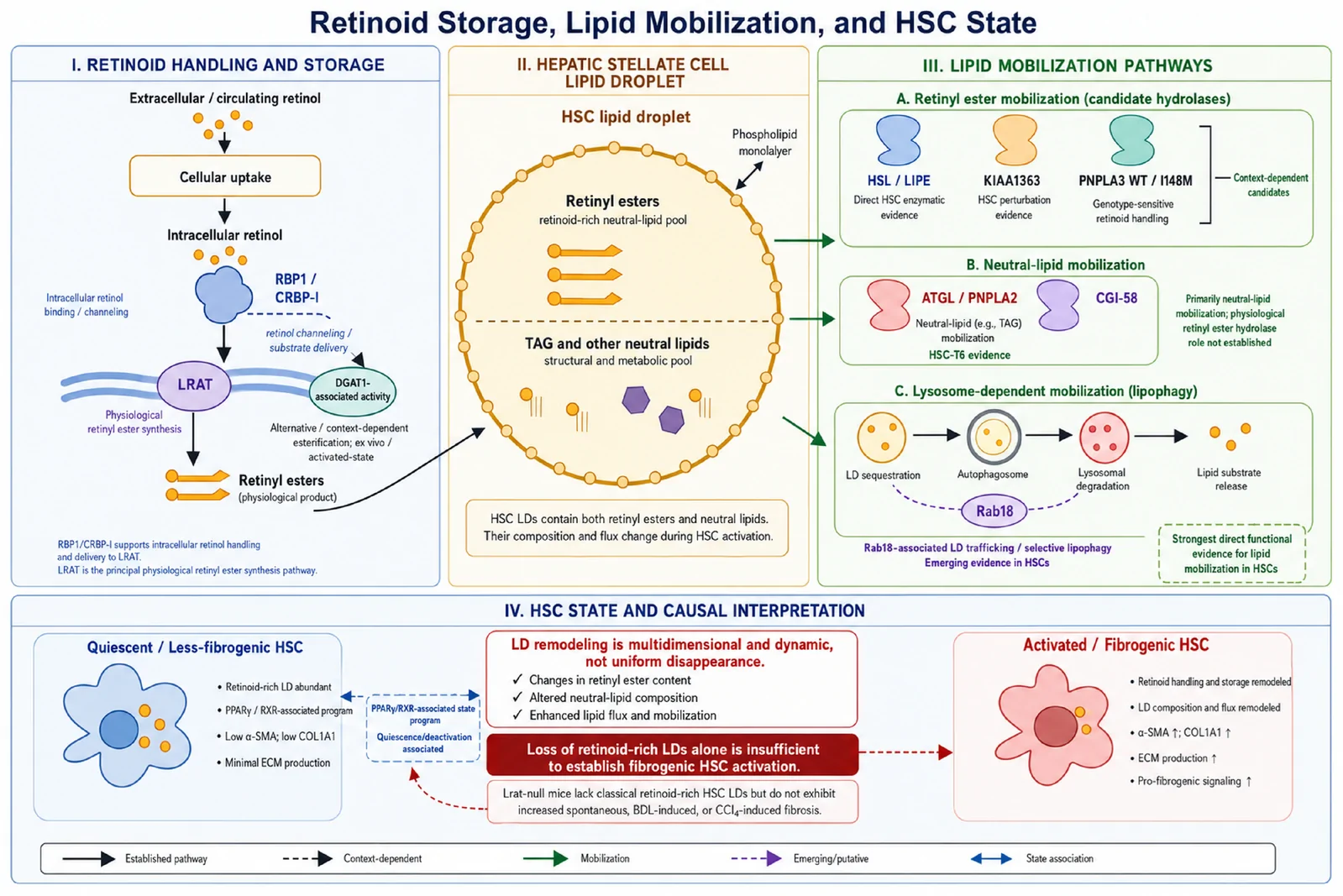
This figure illustrates the processes of retinoid storage, lipid mobilization, and the state of hepatic stellate cells (HSCs). The intracellular handling of retinol is facilitated by RBP1/CRBP-I, which directs retinol toward physiological retinyl ester synthesis mediated by LRAT. In contrast, esterification associated with DGAT1 is depicted as context-dependent. Candidates implicated in retinyl ester mobilization, including HSL/*LIPE*, KIAA1363, and PNPLA3, are differentiated from the ATGL/*PNPLA2*–CGI-58 neutral lipid system characterized in HSC-T6 cells, as well as from lysosome-dependent autophagy and lipophagy pathways. Rab18 is identified as an emerging regulator involved in lipid droplet trafficking and selective lipophagy. Regulatory programs associated with PPARγ/RXR are portrayed as modulators of cell state rather than as enzymes responsible for retinyl ester storage. Data derived from *Lrat*-null models of fibrosis establish a critical causal boundary, showing that loss of classical retinoid-rich HSC LDs did not cause spontaneous fibrosis or exacerbate fibrosis in the injury models tested [29]. WT, wild type. Red dotted arrows indicate contextual relationships rather than direct causal mechanisms; the curved arrow highlights evidence from Lrat-null models showing that loss of retinoid-rich lipid droplets alone is insufficient to establish fibrogenic HSC activation. Upward arrows indicate increased expression. Created using ChatGPT (OpenAI, GPT-5.6).

**Figure 3:**
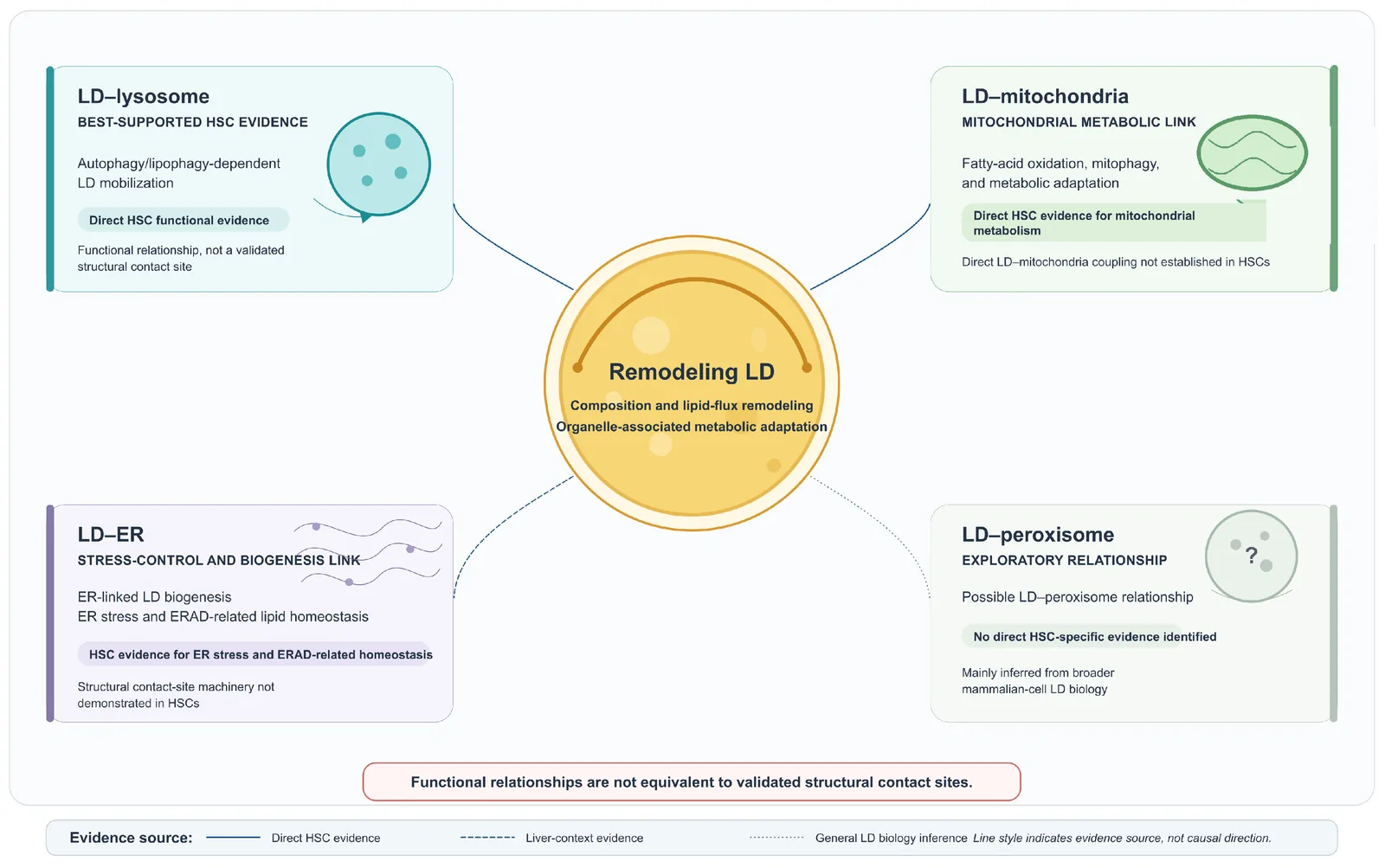
This figure illustrates an evidence-tiered framework of organelle interactions involved in the remodeling of lipid droplets within hepatic stellate cells (HSCs). The association between lipid droplets (LDs) and lysosomes is supported by the most robust direct functional evidence in HSCs, primarily through autophagy- and lipophagy-mediated lipid mobilization. This functional evidence does not establish the existence of a stable LD–lysosome contact site in HSCs. Investigations focusing on HSCs provide evidence for mitochondrial roles in metabolic processes and quality control during cellular activation, yet they do not confirm direct lipid transfer between LDs and mitochondria nor the necessity of a structural contact interface. Similarly, studies indicate that endoplasmic reticulum (ER) stress and ER-associated degradation (ERAD) pathways contribute to lipid homeostasis in HSCs, although direct tethering or contact-site dependence between LDs and the ER specific to HSCs has not been established. The relationship between LDs and peroxisomes in this context remains largely unexplored. In the figure, solid, dashed, and dotted lines represent evidence derived from direct HSC studies, broader liver-context research, and general lipid-droplet biology, respectively; the style of the lines denotes the source of evidence rather than implying causality. The dashed LD–ER connector denotes liver-context support for the broader relationship, whereas the box separately identifies direct HSC evidence for ER stress and ERAD-related homeostasis, not an established LD–ER contact mechanism. Created using ChatGPT (OpenAI, GPT-5.6).

**Table 1:** Key Regulators and Processes Involved in Hepatic Stellate Cell Lipid-Droplet Remodeling: Evidence, Experimental Models, and Interpretive Limitations.

Regulator/Process	Evidence Basis	Experimental Model	Interpretation	Key Limitation
RBP1 (CRBP-I) [27,28]	Genetic evidence for retinoid storage	*Rbp1*-null mice; HSC-relevant liver storage	Channels intracellular retinol toward efficient esterification and storage	Whole-liver/genetic evidence; not a stand-alone activation switch
LRAT [29,30]	Genetic storage evidence and direct HSC lipidomics	*Lrat*-null mice; primary HSCs	Principal physiological retinyl esterification machinery for classical retinoid-rich HSC LDs	Loss of LRAT changes storage identity but does not itself establish fibrosis
HSL/*LIPE* [31]	Direct enzymatic HSC evidence	Quiescent human and rat HSCs	Candidate retinyl ester hydrolase	Physiological contribution across injury states remains unresolved
KIAA1363 [32]	Direct perturbation evidence	Cultured murine and human HSCs	Modulates retinyl ester turnover	Culture-model dependence and substrate scope limit generalization
PNPLA3 [33,34,35]	Direct genotype and human-HSC evidence	Human HSCs; primary 3D models	Genotype-dependent retinyl-palmitate handling and fibrogenic phenotype	I148M effects cannot be generalized to all LD turnover
ATGL/*PNPLA2* [36]	LD localization and cell-line evidence	HSC-T6	Neutral-lipid mobilization candidate	Not established as a physiological HSC retinyl ester hydrolase
PLIN5; PPARγ/RXR-associated programs [9,10,37,38,39,40]	Cell-state regulation	Activated HSCs and experimental reversion models	Links LD organization to quiescence/deactivation-associated transcriptional state	Neither is a retinyl ester storage enzyme
Rab18 [41,42]	Emerging selective-lipophagy evidence	Experimental HSC lipophagy models	Candidate regulator of LD trafficking and lipophagic flux	Limited studies; not a universal HSC mechanism

**Table 2:** Evidence-tiered interpretation of LD–organelle relationships in HSCs.

Axis	Direct HSC Evidence and Boundary	Liver-Context Evidence	General LD Biology	Working Conclusion
LD–lysosome	Autophagy-dependent lipid mobilization has direct functional support; structural contact sites are not equivalently established [43,44]	Liver injury and fibrogenesis context (reviews) [7,60]	Lysosomal LD processing is well characterized [21,22,45]	Strongest functional HSC relationship; separate flux from contact-site claims
LD–mitochondria	Direct HSC evidence supports CPT1A-dependent fatty-acid oxidation and mitochondrial adaptation during activation; direct LD-to-mitochondria substrate transfer or contact-site dependence has not been established [48,53].	MASH and injury metabolism [8,54,55,61]	Physical contacts occur in other cell types [56]	Mitochondrial metabolic involvement is supported; direct HSC LD–mitochondria coupling remains unproven.
LD–ER	HSC studies support ER stress and ERAD-associated lipid-homeostasis regulation [57,58]; direct HSC-specific LD–ER tethering or contact-site dependence has not been demonstrated.	Hepatic stress biology [7,8,60]	ER is central to LD biogenesis in general LD biology [18,19,20,21,22]	HSC ER-stress and lipid-homeostasis evidence is stronger than evidence for a direct LD–ER contact mechanism.
LD–peroxisome	No direct HSC-specific evidence identified	Limited indirect context	Contact/metabolic relationships occur elsewhere [59]	Exploratory HSC axis; no causal or structural conclusion

**Table 3:** Mechanism-organized therapeutic strategies related to HSC lipid-droplet remodeling.

Therapeutic Node	Translational Position	Mechanism/Rationale	Evidence Basis	Major Limitation/Caution
Retinoid handling	Mechanistic preclinical target	Restore defined storage/signaling states rather than LD number alone	Genetic, cellular and liver-context evidence [27,28,29,30]	Storage loss alone is not a fibrosis-causality test
PPARγ-associated phenotype restoration	Preclinical phenotype strategy	Supports quiescence/deactivation-associated programs	Direct HSC experimental support [9,10,37]	Systemic receptor effects and state dependence
Lipophagy/lysosomal mobilization	Mechanism-led preclinical target	Modulates lipid mobilization that can support HSC activation	Direct HSC functional evidence for autophagy/lipophagy [43,44], with supporting preclinical probe studies [41,42,77]	Autophagy has divergent functions across hepatic cell types. Representative probes include curcumol, diallyl trisulfide, and honokiol [41,42,77]; multi-pathway effects limit target attribution.
PNPLA3/genotype-informed strategies	Precision-medicine hypothesis	Addresses genotype-dependent HSC lipid handling	Human HSC and 3D-model evidence [33,34,35]	Requires genotype-aware validation and delivery
Mitochondrial metabolism	Metabolic preclinical target	Modulates FAO and activation-associated adaptation	Functional HSC and liver-context evidence [48,53,54,55]	Does not establish LD-contact causality
ER stress/lipid homeostasis	Preclinical stress-response strategy	Modulates ER stress and ERAD-linked lipid homeostasis	Early preclinical evidence [57,58]	Arctigenin probes ERAD-linked homeostasis [57]; oroxylin A separately probes ATGL-dependent LD turnover [79], not ERAD. Natural-product pleiotropy and limited human validation remain key limitations.

## Data Availability

No new data were created or analyzed in this study. Data sharing is not applicable to this article.

## Abbreviations

ABHD5, alpha/beta hydrolase domain-containing 5; ACC1, acetyl-CoA carboxylase 1; AMPK, AMP-activated protein kinase; ATGL, adipose triglyceride lipase; BDL, bile duct ligation; CCl4, carbon tetrachloride; CEACAM1, carcinoembryonic antigen-related cell adhesion molecule 1; CGI-58, comparative gene identification-58; COL1A1, collagen type I alpha 1 chain; CPT1A, carnitine palmitoyltransferase 1A; CRBP-I, cellular retinol-binding protein I; CRTC2, CREB-regulated transcription coactivator 2; DGAT1, diacylglycerol O-acyltransferase 1; ECM, extracellular matrix; ER, endoplasmic reticulum; ERAD, endoplasmic reticulum-associated degradation; ETS1, ETS proto-oncogene 1; FAO, fatty-acid oxidation; HER2, human epidermal growth factor receptor 2; HIV, human immunodeficiency virus; HSC, hepatic stellate cell; HSL, hormone-sensitive lipase; iPSC, induced pluripotent stem cell; LD, lipid droplet; LIPE, lipase E, hormone-sensitive type; LRAT, lecithin:retinol acyltransferase; LSEC, liver sinusoidal endothelial cell; MASLD, metabolic dysfunction-associated steatotic liver disease; MASH, metabolic dysfunction-associated steatohepatitis; PGC-1α, PPARγ coactivator 1α; PINK1, PTEN-induced kinase 1; PLIN5, perilipin 5; PNPLA2, patatin-like phospholipase domain-containing 2; PNPLA3, patatin-like phospholipase domain-containing 3; PPARα, peroxisome proliferator-activated receptor alpha; PPARγ, peroxisome proliferator-activated receptor gamma; RBP1, retinol-binding protein 1; RXR, retinoid X receptor; TAG, triacylglycerol; α-SMA, alpha-smooth muscle actin.
